# P-1155. Activity of Ceftolozane/Tazobactam, Imipenem/Relebactam and Comparators Against non-Morganellaceae Enterobacterales and Pseudomonas aeruginosa Isolated from Patients with Invasive Respiratory Infections: SMART United States, 2021-2023

**DOI:** 10.1093/ofid/ofaf695.1348

**Published:** 2026-01-11

**Authors:** Mark G Wise, Karri A A Bauer, John Esterly, Fakhar Siddiqui, Katherine Young, Mary Motyl, Daniel F Sahm

**Affiliations:** IHMA, Schaumburg, IL; Merck & Co, Inc, Kenilworth, New Jersey; Merck & Co., Inc., Rahway, New Jersey; Merck & Co., Inc., Rahway, New Jersey; Merck, Rahway, New Jersey; Merck, Rahway, New Jersey; IHMA, Schaumburg, IL

## Abstract

**Background:**

Gram-negative bacteria can be the cause of life-threating invasive respiratory tract infections (RTIs), including pneumonia, tracheobronchitis, and lung abscesses. Imipenem/relebactam (IMR) is a combination of imipenem with the β-lactamase inhibitor relebactam. Ceftolozane/tazobactam (C/T) combines ceftolozane, an anti-pseudomonal cephalosporin, with tazobactam. We evaluated the activity of IMR, C/T and comparators against isolates of non-Morganellaceae Enterobacterales (NME) and *Pseudomonas aeruginosa* (PA) that were collected in the US for the SMART surveillance program (2021-2023) from patients with invasive and non-invasive respiratory samples stratified by length of hospital stay (< 48h [presumed community-acquired infections] vs. ≥48h [presumed hospital-acquired infections]).

**Figure:**
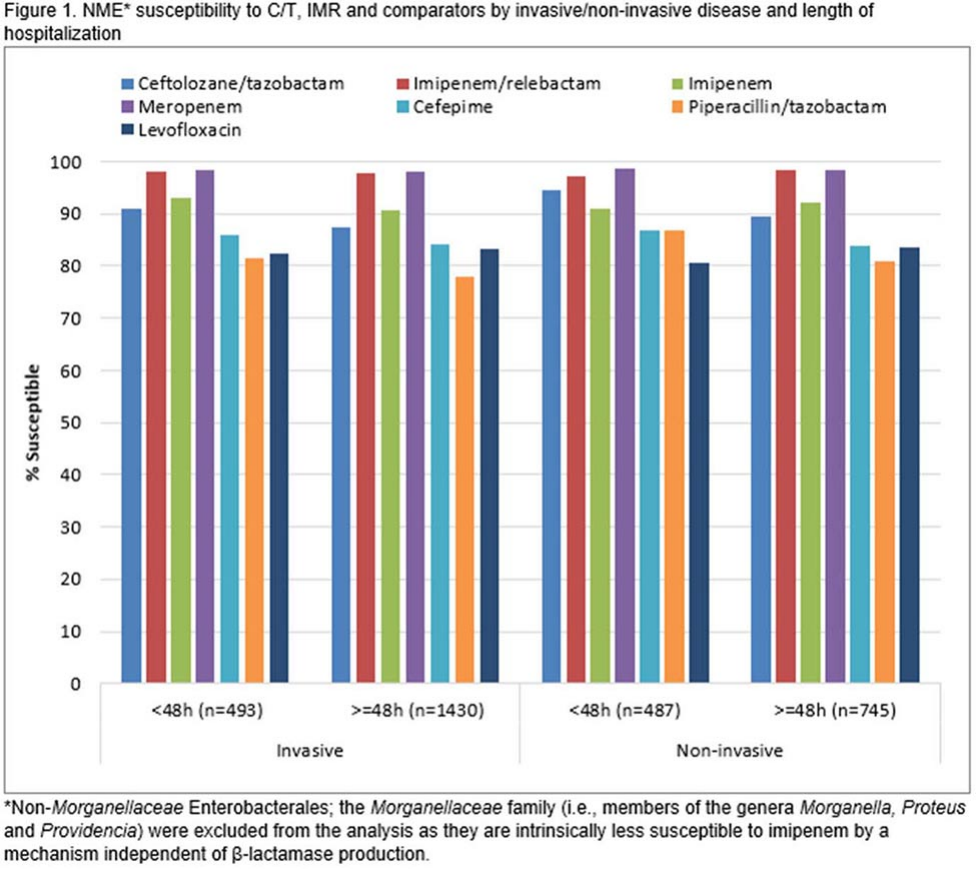

**Figure:**
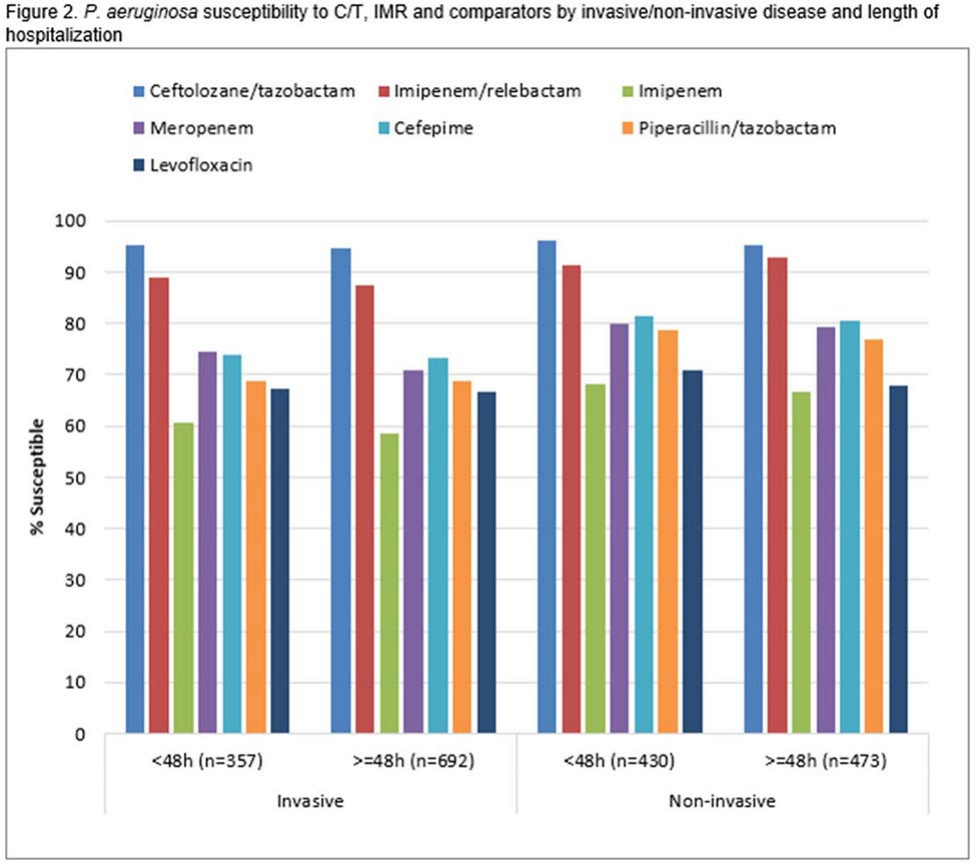

**Methods:**

In 2021-2023, 27 sites in the US collected up to 100 Gram-negative pathogens per year from patients with RTIs. Invasive vs. non-invasive disease categories were based on sampling source (invasive: bronchial brushing, bronchoalveolar lavage, endotracheal aspirate, lungs, thoracentesis; non-invasive: sputum or other). MICs were determined via CLSI broth microdilution and interpreted with 2025 CLSI criteria. Among Enterobacterales, only NME were considered as the *Morganellaceae* display intrinsic reduced susceptibility to imipenem by a mechanism other than β-lactamase production.

**Results:**

Susceptibility to commonly used β-lactams was generally lower among NME from presumed hospital-acquired infections compared to community-acquired (Fig. 1). No clear pattern was linked to invasive vs. non-invasive infections. IMR and meropenem were the most active agents in all categories, inhibiting >97% of the NME. Among PA, isolates from invasive samples consistently displayed lower susceptibility than those from non-invasive infections, regardless of length of hospital stay (Fig. 2). C/T was the most active agent in all categories (inhibiting ≥94.7%) followed by IMR (≥87.4% inhibited).

**Conclusion:**

IMR is an important treatment option for patients with respiratory tract infections caused by NME, while C/T was the most active agent against *P. aeruginosa* causing RTIs, regardless of length of stay or invasive disease.

**Disclosures:**

Mark G Wise, PhD, IHMA: Employee Karri A. A. Bauer, PharmD, Merck: Employee Fakhar Siddiqui, MD, MBA, Merck & Co Inc.: Stocks/Bonds (Public Company) Katherine Young, M.S., Merck & Co., Inc.: Stocks/Bonds (Public Company) Mary Motyl, Ph.D., Merck and Co., Inc.: employee

